# Maternal *Toxoplasma gondii* Infection Perturbs Foetal and Maternal Foetal Interface Metabolism, Exposing the Foetus to Kynurenine

**DOI:** 10.3389/bjbs.2025.14989

**Published:** 2026-02-04

**Authors:** Hafiz Arshad, Gareth Westrop, Natércia Teixeira, Margarida Borges, Craig W. Roberts

**Affiliations:** 1 Strathclyde Institute of Pharmacy and Biomedical Sciences, University of Strathclyde, Glasgow, United Kingdom; 2 Associate Laboratory i4HB-Institute for Health and Bioeconomy, Faculty of Pharmacy, University of Porto, Porto, Portugal; 3 UCIBIO-Applied Molecular Biosciences Unit, Laboratory of Biochemistry, Department of Biological Sciences, Faculty of Pharmacy, University of Porto, Porto, Portugal

**Keywords:** kynurenine, metabolomics, liquid chromatography-mass spectrometry, placental tissue, decidua, *Toxoplasma gondii*, Toxoplasmosis

## Abstract

**Introduction:**

*Toxoplasma gondii* infection during pregnancy can result in abortion or congenital infection. Events in the maternal-foetal interface, which form a selective barrier between the maternal and foetal circulations and where critical immunological adaptations occur, are critical in determining the pregnancy outcome. Recent studies have demonstrated that *T. gondii* infection can alter host metabolism, but how *T. gondii* infection alters the placenta or the foetus metabolome has not been reported.

**Methods:**

Herein, for the first time, we use liquid chromatography mass spectrometry (LCMS) in the BALB/c murine model of congenital *T. gondii* to address this shortcoming.

**Results:**

Maternal infection resulted in dysregulation of free amino acids with significant decreases in the levels of arginine, proline, threonine, methionine, leucine, glycine and glutamine detected in the decidua. Similar changes were noted in the placenta, although differences were less pronounced. In contrast, amino acid levels were not significantly altered in the foetal extracts. Results demonstrate that *T. gondii* infection induces the highest number of metabolite changes in the maternal serum. However, a subset of these changes was also found in the maternal-foetal interface and in the developing foetus. Maternal infection resulted in changes to arginine metabolism and downregulation of the urea cycle**.** Specifically, ornithine, arginosuccinate and citrulline were significantly decreased in all three tissues following maternal infection. Increased levels of spermidine were evident in the placenta and foetal extracts and not in the decidua from maternally infected mice. This indicates that maternal *T. gondii* infection downregulates the urea cycle, while increasing flux into polyamine biosynthesis in the decidua, placenta and foetus. Maternal infection resulted in an alteration to the tryptophan degradation pathway. Significantly decreased levels of kynurenine were seen in the decidua and placenta of maternally infected mice in comparison with the uninfected controls. In contrast, there was a significant increase in kynurenine in foetal extracts from maternally infected mice. Some metabolites from microbiome origin, including indoxylsulfate and 4-guanidinobutanoate, were changed compared with the controls, suggesting the potential of *T. gondii* to change the host microbiome.

**Discussion:**

The data presented herein demonstrate that *T. gondii* infection during pregnancy alters the metabolome of the maternal-foetal interface and developing foetus. Notably increased kynurenine and decreased tryptophan levels were found in the foetal tissue. As kynurenine is known to be produced during maternal immune activation and has been implicated in the development of psychoneurological diseases these changes could have important implications for the offspring over their lifetime.

## Introduction

*Toxoplasma gondii* is an intracellular parasite that can infect humans and all warm-blooded animals, including birds and mammals. Although disease manifestations are normally self-limiting in immunocompetent humans, serious or even fatal disease can occur in immunodeficient individuals. This parasite is also a risk during pregnancy, where it can cause foetal loss, pre-term birth or congenital infection. In humans, congenital infection is acquired through the placenta and arises from primary acquired maternal infection during gestation [1]. Vertical transmission frequency and foetal damage severity depend on the pregnancy stage when maternal infection occurs. Infection during the first trimester is more likely to cause abortion, whereas infection in the third trimester is more likely to result in vertical transmission [2–4]. The severity and frequency of this infection also depend on which stage of gestation the mother is infected. Early infection results in the most severe congenital disease, while late infection results in mild or even asymptomatic infection at birth. Women with chronic *T. gondii* infections rarely transmit the disease to their foetuses. Likewise, it has been shown that vertical transmission only occurs in BALB/c mice infected for the first time during pregnancy [5, 6].

As an intracellular parasite, *T. gondii* has evolved a close relationship with their mammalian host cells and their metabolisms are inextricably linked [7, 8]. Some recent studies have demonstrated that *T. gondii* remodels host cell metabolism, including alterations to mitochondrial and glycolytic functions. In addition, *T. gondii* is known to rely on host cells’ metabolites, including arginine, tyrosine, tryptophan, purines and cholesterol [9–11]. However, the interaction of *T. gondii* at the maternal-foetal interface and its impact on the success of pregnancy in the cases of congenital transmission have not been studied. The maternal foetal interface comprises the decidua (maternal adapted uterus) and the foetal-derived placenta. The decidua supplies oxygen and nutrients to the placenta. The placenta regulates and optimises nutrient transport, although maternal status can affect the availability of these metabolites. The placenta regulates and supplies nutrients (including glucose, amino acids, fatty acids and cholesterol) to the foetus, influenced by foetal communication through growth factors and nutrient use. Maternal illness, including infection, can influence placental metabolism and function, resulting in disruption of optimum nutrient transfer [12].

The following studies were undertaken using a BALB/c murine model of congenital toxoplasmosis previously described, to determine the influence of maternal *T. gondii* infection on the metabolism of the decidua, placenta and foetus [13]. To the best of our knowledge, this study covers the first comprehensive metabolomic profiling of the maternal foetal interface, developing foetus and maternal serum.

## Materials and Methods

### Metabolomics

#### Parasite

ME49 strain of *T. gondii* was kindly provided by Dr Marcus Meissner (Parasitology department of the Faculty of Medicine, Heidelberg University, Germany). Viable tachyzoites were obtained by *in vitro* infection of Human Fosenskin Fibroblasts (HFF from ATCC® SCRC-1041™); cultivated in Dulbecco’s Modified Eagle Medium (DMEM) supplemented with L-glutamine (2 mmol/L), sodium pyruvate (1 mmol/L), foetal bovine serum (10%), 100 U/mL penicillin and 100 μg/mL streptomycin (all from Life Technologies, Europe). Cell cultures were maintained in flasks at 37 °C, in a humidified atmosphere of 5% CO_2_. For *in vitro* parasite maintenance, a 1:10 ratio (HFF cells: parasite) was used. When the majority of fibroblasts exhibited multiple intracellular tachyzoites, namely in rosettes, they were detached using a cell scraper and disrupted using a 25G needle and syringe 10 times. The cell suspension was filtered using a 5-µm filter (Sartorius) and centrifuged at 500 *g* for 6 min at room temperature to eliminate HFF cell debris. Tachyzoites were counted using a Neubauer chamber.

### Mice and Infection

Female BALB/c mice were obtained from Charles River (L'Arbresle, France) and maintained in the Animal facility of the Institute of Biomedical Sciences Abel Salazar (Porto, Portugal). BALB/c females, aged 8–12 weeks, were mated with fertile males (1 male/ 2 females/ cage). Day 1 of pregnancy was set as the day when vaginal plug became apparent. The mice were fed with a sterilised, standardized commercial rodent diet in the pellet form composed by: crude protein: 18%–24%; Crude fat: 4%–6%; Crude fiber: 4%–6%; essential vitamins (A, D3, E, K, B-complex, folic acid, biotin) and minerals (calcium, phosphorus, sodium, potassium, magnesium, iron, zinc, copper, manganese, iodine, selenium). Diet and water are provided *ad libitum.* All pregnant mice obtained were primiparous. Mice were intraperitoneally (i.p.) infected with 5 × 10^3^ viable tachyzoites of the *T. gondii* Me49 strain on day 7 of gestation. The mice were weighed at day 0 of infection, and their 20% cut-off body weight was calculated as a standard procedure, determining the baseline for the severity of distress and pain of animals used in experimental research. As soon as the mouse reached this 20% cut-off value, it was sacrificed to avoid further suffering and discomfort. Taking this into consideration, infected animals were sacrificed on either day 13 or day 14 of pregnancy (6 or 7 days post-infection). and the uterine horns were collected. The implantation units were removed from the uterine horns and dissected: the foetus and the foetal portion of the placenta were separated from maternal tissues by blunt dissection [14]. As previously described, no alteration in the resorption rate was observed between infected pregnant and control pregnant mice in our experimental conditions [13]. The resorted units were not used in the dissection, since it wasnot possible to observe the integral tissues. The maternal blood collection was performed after the induction of general anaesthesia in the animal with isoflurane. Control groups corresponded to uninfected animals at day 13 or day 14 of pregnancy. The sacrifice of animals consisted of anaesthetic induction with 5% isoflurane, followed by cervical dislocation. All procedures involving animals were performed in accordance with the European Convention (ETS 123), the EU Directive of 22 September 2010, and Portuguese law (DL 113/2013) concerning the protection of animals used for scientific purposes. The authorisation to perform the experiments was issued by the Organ Responsible for Animal Welfare (ORBEA) at ICBAS (document 315/2019/ORBEA) and the responsible national board authority, Direção-Geral de Alimentação e Veterinária (document 024143). Samples were also collected from uninfected mice at day 13 or 14 days of pregnancy (controls).

### Tissue Extracts

For the metabolomic profiling, two experiments were performed; each experimental set included a group of uninfected pregnant animals and a group of infected pregnant animals. The total number of animals used for each group and experiment is detailed in Supplementary Table S1. The studies were designed to focus mainly on the metabolomics profile of the maternal foetal interface (decidua and placenta) and of the developing foetus. Hence, the tissues were dissected and metabolites extracted for comparative investigation. Initially, the metabolomic profile was studied in the maternal-foetal interface and foetus, and subsequently, the main focus shifted to the developing foetus and maternal serum. All samples from these tissues were extracted and placed in ice-cold 0.1% formic acid before proceeding with the metabolite extraction procedure.

### Metabolite Extraction

Extraction of polar and non-polar metabolites was achieved using a two-step extraction protocol with chloroform, methanol and deionised water. The tissues were collected in a microtube containing 400 µL 0.1% formic acid. The microtubes were weighed before and after tissue collection to determine the tissue weight. Tissue samples were removed from the formic acid solution and added to a pre-chilled 15 mL polypropylene tube containing 4 mL/g tissue sample of ice-cold methanol (Fisher, Optima LC/MS grade) and 0.85 mL/g ice-cold deionised water. Homogenisation of the tissue sample was carried out, keeping the sample chilled in an ice bucket. 4 mL/g cold chloroform (Fisher chemicals) and 2 mL/g dH_2_O were then added, and the homogenate was vortexed for 60 s. Samples were allowed to partition on ice for 10 min. After this, samples were centrifuged at 12000 *g* for 15 min at 4 °C to produce a biphasic mix. The upper polar layer was removed from each extract and transferred to a 1.5 mL HPLC vial with a 200 μL insert. Metabolites are present in the upper layer, while the lower layer contains mostly extraction solvents. For the maternal serum extraction, serum sample (75 μL) was mixed with the 300 μL of the extraction buffer (1 volume chloroform: 3 volumes methanol). After shaked in a thermomixer for 1 H at 4 °C, the samples were centrifuged at 12,000 *xg* for 10 min at 4 °C and 200 μL of supernatant were transferred to a HPLC vial with a 200 μL insert. All the samples were then stored at −80 °C. The samples were dispatched on dry ice to the University of Strathclyde, where these samples/extracts were received and stored at −80 °C, before metabolomics study using the LCMS technique.

### LCMS Analysis

Samples from the first experiment were analysed at the University of Strathclyde, Glasgow, UK using an LCMS platform that consisted of an Accela 600 HPLC system in combination with an Exactive Orbitrap mass spectrometer (Thermo Fisher Scientific, Bremen, Germany). Two columns, with complementary abilities were used, zwitterionic ZIC- pHILIC column (150 mm × 4.6 mm; 3.5 μm, MerckSequant, Gillingham, UK) and the reverse phase ACE C18- PFP column (150 mm × 4.6 mm; 3.5 μm, Hichrom, Theale, UK), with an injection volume of 10 μL and a flow rate of 0.3 mL/min. A gradient of mobile phase A, 20 mM ammonium carbonate pH 9.2, and mobile phase B, acetonitrile (I) was used to elute the ZIC- pHILIC column. The concentration of buffer A was increased from 20% to 80% over 30 min and then maintained at 92% for 5 min, before equilibrating at 20%. The mobile phases for the reverse phase column consisted of: A, 0.1% (v/v) formic acid in H2O; B, 0.1% (v/v) formic acid in acetonitrile (can). Buffer A was decreased from 95% to 10% over 30 min and maintained at 10% for 5 min. LCMS for samples from the second experiment were carried out by Glasgow Polyomics, University of Glasgow UK. This used the same ZIC-pHILIC Column on a Dionex Ultimate 3000 RSLC system coupled with a Thermo Scientific QExactive Orbitrap mass spectrometer (Thermo Fisher Scientific, Hemel Hempstead, United Kingdom). The injection volume was 10 μL and the flow rate was 0.3 mL/min. The elution gradient for the ZIC-pHILIC column was the same as described above.

### Data Retrieval

The output from the LCMS are Raw files containing positive and negative modes, and were transferred to a server to convert into csv files. Raw data files were first converted to the mzXML format using msconvert (ProteoWizard), which splits the data according to the polarity. Peaks in each file were identified and extracted by XCMS to generate a peakML file and peaks from replicate samples were aligned using mzMatch.R. All peaks from replicate samples were combined in a single peakML file. This file was converted to a csv file containing retention times, accurate masses and peak intensities for each peak in all the samples. This csv file was imported into IDEOM v19 for metabolite identification [15]. Data generated are available at https://doi.org/10.15129/b313b637-48e3-46bb-bd37-3a25f396274d.

### Confirmation of Metabolite Identification

The identity of the metabolites was confirmed using the ToxID 2.1 software (Thermo Fischer Scientific Inc., Hemel Hempstead, UK) to compare metabolites’ relative abundance against retention time and *m/z* mass. Many metabolites were compared with their authentic standards regarding their relative abundance against retention time and *m/z* mass.

### Software

All the data obtained were subjected to further statistical analysis for multivariate analysis in a software called SIMCA® version 16.0.1 (Umetrics, Sweden). For an overview of the data, Principal component analysis (PCA) and Orthogonal partial least squares discriminant analysis (OPLS-DA) were chosen. Data were mean centered and pareto scaled. Another software, MetaboAnalyst version 5, was used for further features, including volcano plots to determine variables and their significance based on log 2FC and–log10 P. For metabolomics data, a Student’s t-test was used for all identified metabolites. Significance threshold was set to p < 0.05. GraphPad Prism was used to plot the graphs. Throughout this study, *p < 0.05, **p < 0.01, ***p < 0.001.

## Results

### Decidua, Placenta and Developing Foetus Undergo Distinct Metabolic Alterations

**FIGURE 1:**
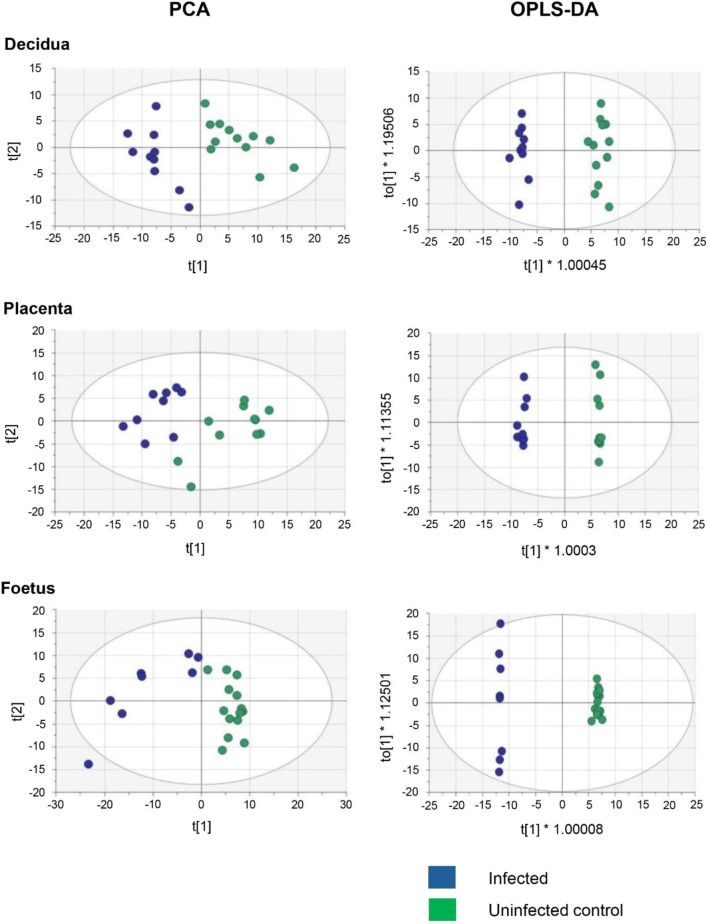
Multivariate Analysis of decidua, placenta, and developing foetus metabolism from *T. gondii*-infected day-14 pregnant mice (infected; 7 days post-infection) or uninfected day-14 pregnant mice using a pHILIC column. Each dot represents an individual tissue. A total of 13 deciduas, 13 placentas and 14 foetuses from uninfected control animals and 10 deciduas, 10 placentas and 8 foetuses from infected animals. The data was analysed by a cross-validated orthogonal partial least square discriminant approach (OPLS-DA).

**FIGURE 2:**
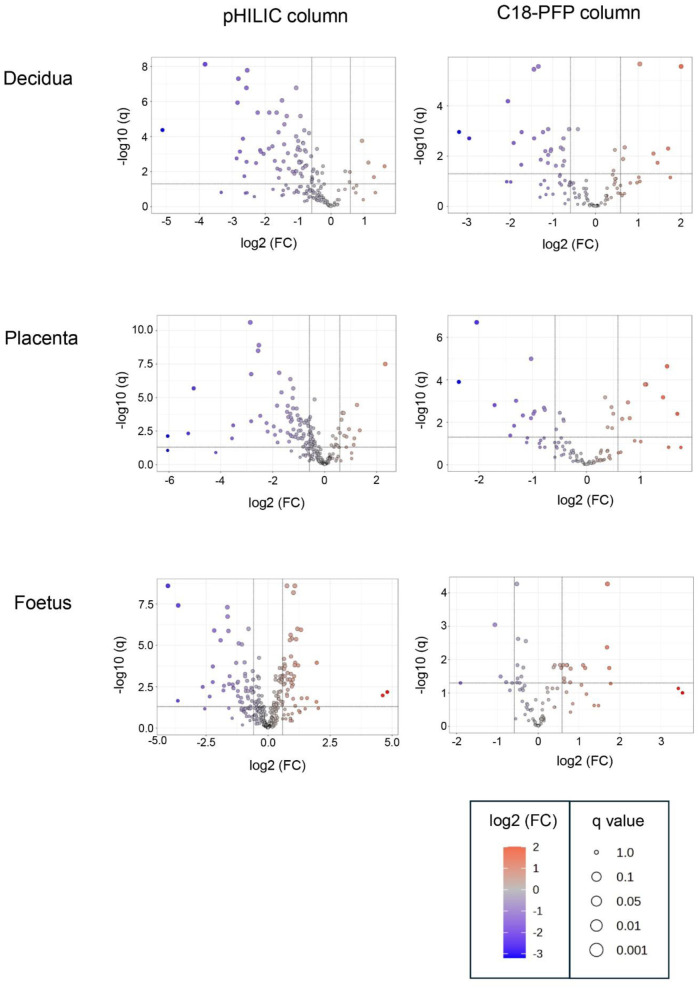
Volcano plots for the analysis of significant metabolites in decidua, placenta and developing foetal extracts obtained from *T. gondii*-infected day-14 pregnant mice using the pHILLIC and C-18 PFP column. The X-axis shows fold change (log2 FC) in *T. gondii*-infected day-14 pregnant mice relative to uninfected day-14 pregnant mice, and the Y-axis is indicative of significance (−log10 q). Significantly increased/decreased metabolite have log2 FC thresholds of 0.6 (or a 1.5-fold increase) and −0.6 (or a 1.5 fold decrease) and −log10 q ≥ 1.3 (or an FDR adjusted p ≤ 0.05). These are represented by dots located in the upper right and upper left rectangles respectively. All other metabolites are below the threshold for significance.

The objective of this study was to determine if *T. gondii* infection alters the metabolism of the maternal-foetal interface and the foetus. LCMS, using a pHILIC column for the separation step, identified 162 metabolites in decidua extracts, 217 in placenta extracts and 322 in foetal extracts. To extend the range of metabolites detected, LCMS was repeated using a C18-PFP column, with 107 metabolites identified in the decidua samples, 92 in the placenta and 93 in the foetus. Principal component analysis (PCA) of the output from the pHILIC column separated the decidua, placenta, and foetal extracts into infected and uninfected groups, based on the peak intensity values of the metabolites (Figure 1). A cross-validated orthogonal partial least square discriminant analysis (OPLS-DA) also showed clear separation of the infected and uninfected samples, although the foetal samples from the infected group appeared to form two separate clusters (Figure 1). Score plots for PCA and OPLS-DA for data from the C18-PFP, separated infected and control groups in the same way (data not shown). OPLS-DA ranks the metabolites by a Variable Importance for the Projection (VIP) scores, with a value ≥1.0 indicating that the metabolite profile makes an essential contribution to the difference between the infected and uninfected groups. Peak intensities were used to calculate fold-change (FC) (infected/uninfected) and q-values (False Discovery Rate corrected p-values) for each metabolite. Figure 2 shows volcano plots of log2 FC against–log10 q, with significant changes identified as Log 2 FC ≤ −0.6 or ≥0.6 and −log10 q ≥ 1.3. The log2 FC threshold of 0.6 represents a 1.5-fold change, and a −log10 q value of 1.3 is 0.05. Table 1 shows the number of significantly altered metabolites identified in each column in the different tissues. The importance of these metabolites is indicated by a VIP value ≥0.8, and the majority show a VIP ≥1.0. All metabolites identified with high confidence are shown in their relevant tissue sections along with their formula, mass, retention time, log2 fold change and−log10 p and −log10 q values in Supplementary Tables S2–S7. Furthermore, no statistically significant differences in the weight of the tissues collected were achieved between control pregnant mice and infected pregnant mice (Supplementary Table S8).

**TABLE 1:** Numbers of metabolites identified in extracts from decidua, placenta and foetus.

Column used	Decidua				Placenta			Foetus		
	All	Up	Down	All	Up	Down	All	Up	Down
pHILIC	128	7	54	167	10	44	267	40	54
C18-PFP	73	10	18	43	3	7	38	10	2
Both	34	1	14	49	4	19	55	8	4
Total	235	18	86	259	17	70	360	58	60

All, total number of metabolites. Up, metabolites that showed significantly increased levels in extracts from *Toxoplasma* infected mice, compared with uninfected controls (Log2 FC, ≥ 0.6, −log10 p, ≥ 1.3). Down, metabolites that show decreased levels (log2 FC, ≤ −0.6, −log10 p, ≥ 1.3). Values shown for metabolites detected with the pHILIC column only, the C18-PFP column only or with both columns.

### Maternal *T. gondii* Infection Induces Alterations in Metabolic Pathways in the Maternal-Foetal Interface and in the Developing Foetus

A careful analysis of the data from the two columns identified significant alterations in the decidua, placenta and foetus extracts following maternal infection. These changes mainly affected free amino acid levels and pathways involved in arginine, tryptophan and nicotinamide metabolism and purine degradation (Table 2; Table 3). The levels of metabolites, including indoxyl sulfate and 4-guanidinobutanoate that were ultimately dependent on the gut microbiome were also significantly decreased (Table 3). Importantly, this study revealed similarities and differences in the way the metabolic profile of each tissue was affected by *T. gondii* infection.

**TABLE 2:** Dysregulation of protein amino acid levels in decidua, placenta and foetus.

Metabolite	Decidua		Placenta		Foetus 1	
	log2 FC	−log10 q	log2 FC	−log10 q	log2 FC	−log10 q
**Arginine**	−0.83	**1.26**	−0.47	**1.26**	−0.02	0.03
**Proline**	−1.05	**2.01**	−0.55	**2.57**	−0.15	**1.26**
**Threonine**	−1.07	**2.02**	−0.53	**2.09**	0.05	0.16
**Lysine**	−0.95	1.12	−0.26	0.75	0.42	**1.77**
**Methionine**	−0.92	**1.88**	−0.59	**2.46**	−0.22	1.19
**Tryptophan**	−1.12	0.79	−0.51	1.32	−0.20	1.14
**Tyrosine**	−1.05	0.92	−0.54	**1.88**	−0.18	0.89
**Phenylalanine**	−1.30	1.03	−0.52	**1.73**	0.45	**2.87**
**Leucine**	−1.11	**1.86**	−0.58	**1.87**	0.13	0.47
**Serine**	−0.99	0.82	−0.23	0.53	0.04	0.12
**Glycine**	−0.88	**1.71**	−0.41	**1.31**	0.43	**2.87**
**Glutamate**	−0.70	1.14	−0.24	0.58	−0.14	1.01
**Glutamine**	−2.84	**5.87**	−0.22	1.03	0.31	**3.14**

Metabolite identities confirmed by matching retention time with an authentic standard are in bold type. FC, fold change (infected/control). Blue shading, more than 1.5 fold decrease in infected samples compared to controls (log2 FC ≤ −0.6). Statistically significant −log10 q values (−log10 p ≥ 1.3) are in bold type.

**TABLE 3:** Dysregulation of metabolic pathways in decidua, placenta and foetus from toxoplasma infected mice.

Metabolite	Column	Pathway	Decidua		Placenta		Foetus	
			log2 FC	−log10 q	log2 FC	−log10 q	log2 FC	−log10 q
Hypoxanthine	C18	1	−1.11	**3.07**	−1.18	**2.32**	−0.79	**6.00**
Xanthosine 5-P	H	1	−1.28	**2.17**	−1.40	**2.57**	−2.35	**1.90**
Xanthosine	H	1	−1.89	**3.32**	−2.53	**6.56**	−2.23	**3.72**
**Xanthine**	H	1	−1.00	**3.75**	−1.04	**2.64**	−0.89	**2.43**
**Urate**	C18	1	−1.23	**2.95**	−1.34	**1.84**	−0.55	0.23
**Allantoin**	C18	1	−0.86	**2.20**	−1.00	**2.46**	−0.11	0.13
**Kynurenine**	H	2	−0.96	**2.58**	−1.08	**1.71**	0.96	**5.41**
**Serotonin**	C18	3	−2.05	**4.18**	​	​	​	​
**Ornithine**	C18	4, 5, 6, 7	−1.34	**5.56**	−1.31	**3.01**	−1.06	**3.04**
**Citrulline**	H	4, 5, 8	−1.88	**5.37**	−1.21	**4.19**	−0.37	**3.00**
Argininosuccinate	H	4, 5	−2.67	**3.88**	−2.48	**2.62**	−1.29	**3.94**
**Spermidine**	C18	4, 6	−0.08	0.09	1.09	**3.78**	1.77	**1.28**
**Spermine**	C18	4, 6	1.45	**1.74**	1.75	0.81	1.37	0.62
Homoarginine	H	9	−1.35	**5.16**	−1.32	**4.81**	−0.21	0.40
**Methylnicotinamide**	H	10	−1.78	**3.44**	−1.42	**3.16**	−0.30	0.78
**Choline**	H	11	−1.06	**4.01**	−0.72	**2.42**	−0.42	**3.13**
Hypotaurine	H	12	−0.94	**2.51**	−0.55	**1.46**	−0.58	**2.18**
**Thiamine**	H	13	−2.23	**5.37**	−1.83	**3.16**	0.00	0.00
**Guanidinobutanoate^a^**	H	14	−2.55	**7.77**	−2.83	**5.11**	−1.63	**6.74**
**Indoxylsulphate^a^**	H	15	−5.17	**4.38**	−4.78	**4.26**	−2.64	**2.49**
TMAO^a^	H	16	−2.81	**7.30**	−2.86	**8.26**	−1.91	**5.31**

^a^ Production of metabolite is dependent on the gut microbiome.

Metabolite identities confirmed by matching retention time with an authentic standard are in bold type. FC, fold change (infected/control). Blue shading, more than 1.5 fold decrease in infected samples compared to controls (log2 FC ≤ −0.6); red shading, 1.5 fold increase in infected samples (log2 FC ≤ 0.6). Statistically significant −log10 q values (−log10 p ≥ 1.3) are in bold type. Blank cells, metabolite not found in metabolomic profile. C18, data from LCMS using a C18 PFP column; H, LCMS using a pHILIC column. Pathways perturbed by infection: 1, purine catabolism; 2, tryptophan degradation (kynurenine pathway); 3, tryptophan metabolism (serotonin biosynthesis); 4, arginine and proline metabolism; 5, urea cycle; 6, polyamine biosynthesis; 7, macrophage activation by T helper type 2 cytokines; 8, macrophage activation by T helper type 1 cytokines; 9, non-protein amino acid produced from arginine and lysine; 10, nicotimamide metabolism; 11, glycerophospholipid metabolism; 12, cysteine metabolism; 13, Thiamine biosynthesis; 14, arginine metabolism–gut microbiome dependent; 15, tryptophan metabolism–gut microbiome dependent; 16, phosphatidylcholine, choline or carnitine metabolism–gut microbiome dependent.

### *T.* gondii Infection Dysregulates Free Amino Acid Levels in the Decidua

Maternal infection significantly decreased the levels of most of the free amino acids detected in the decidua (log 2 FC ≤ −0.6, q ≥ 1.3). These include arginine, proline, threonine, methionine, leucine, glycine and glutamine (Table 2). Glutamate, lysine and phenylalanine followed the same trend, although q values were not significant. In the placenta, levels of several amino acids were decreased, but the differences between the infected and control levels were smaller than in the decidua. In contrast, amino acid levels were not significantly altered in the foetal extracts (Table 2).

### Alteration in Arginine Metabolism and Downregulation of the Urea Cycle

**FIGURE 3:**
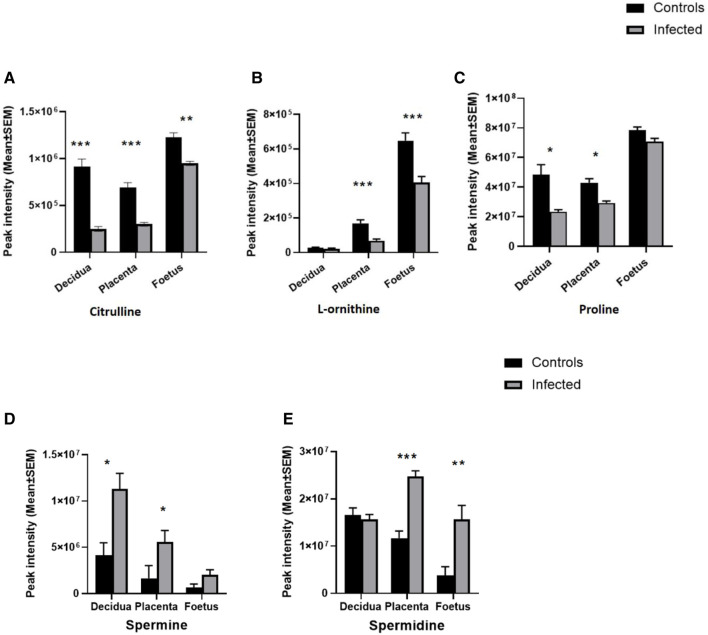
Effect of *T. gondii* maternal infection on the arginine metabolic pathway. Graphs show the peak intensity of important metabolites of arginine detected by LCMS in the decidua, placenta and foetus from uninfected day-14 pregnant mice (Controls) or *T. gondii*-infected day-14 pregnant mice (Infected; 7 days post-infection). **(A)**, citrulline; **(B)**, L-ornithine; **(C)**, proline; **(D)**, spermine and **(E)**, spermidine. The tissues were analysed individually. A total of 13 deciduas, 13 placentas and 14 foetuses from control animals and 10 deciduas, 10 placentas and 8 foetuses from infected animals. Data represent the mean ± SEM. Student’s t-test was performed to determine significance (−log10 q 1.3) where *−log10 q ≥ 1.3, ** −log10 q ≥ 2.3 and *** −log 10 q ≥ 3.3. Data for **(A,C)** were obtained using the pHILIC whereas results for **(B,D,E)** were from the C18-PFP column.

Ornithine, citrulline and argininosuccinate are intermediates of the urea cycle, consisting of a series of reactions in liver hepatocytes that convert ammonia, a toxic product of amino acid deamination, to urea for excretion. Levels of ornithine and arginosuccinate were significantly decreased in all three tissues (Figure 3; Table 2). Citrulline was reduced in the decidua and placenta, with a small but significant change in the foetal extracts (Figure 3; Table 2). Polyamine derivatives of ornithine were identified, with significantly increased levels of spermidine in placenta and foetal extracts, and increased levels of spermine in the decidua (Figure 3; Table 2). There was no change in spermidine levels in the decidua. Although spermine levels were increased in the placenta and foetus, q values were below the threshold for significance (Figure 3; Table 2) The data obtained suggest that *T. gondii* infection results in significant downregulation of the urea cycle and increased flux into polyamine biosynthesis with decreased levels of ornithine and increasing spermidine and spermine in the decidua, placenta and foetus.

### Build-Up of Tryptophan Degradation Products in the Foetus During *T. gondii* Infection

**FIGURE 4:**
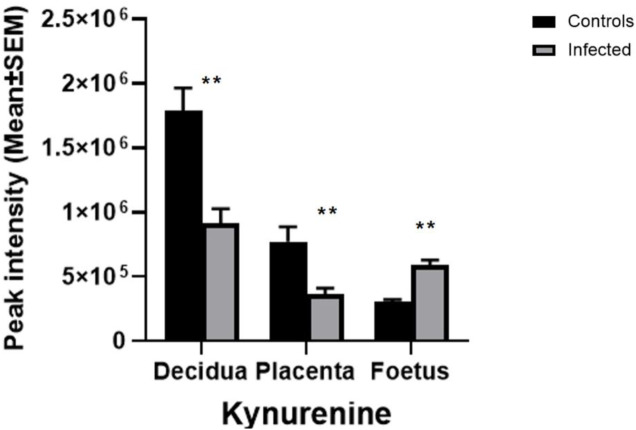
The effect of *T. gondii* maternal infection on the tryptophan degradation pathway. Graphs show peak kynurenine intensity in the decidua, placenta, and foetus from uninfected day-14 pregnant mice (Controls) or *T. gondii*-infected day-14 pregnant mice (Infected; 7 days post-infection) by LCMS analysis. The tissues were analysed individually. A total of 13 deciduas, 13 placentas and 14 foetuses from control animals, and 10 deciduas, 10 placentas and 8 foetuses from infected animals. Data represent the mean ± SEM. Student’s t-test was performed to determine significance (−log10 q = 1.3) where *−log10 q = 1.3, **−log10 q = 2.3, ***−log10 q = 3.3 are significant compared to the control uninfected.

The metabolomic profiles of the maternal-foetal interface in this study indicated *T. gondii*’s ability to alter the tryptophan degradation pathway (Table 3). Significantly decreased levels of kynurenine were seen in the decidua and placenta of maternally infected mice in comparison with the uninfected animals (−log10 q = 2.53 and 1.71). In contrast, there was a significant increase in kynurenine in foetal extracts from maternally infected mice (−log10 q = 5.41). The trend of a change in kynurenine levels in the developing foetus, in the opposite direction from the decidua and placenta, is indicated (Figure 4). Together, these results demonstrate the build-up of tryptophan degradation products in the foetus during *T. gondii* infection.

In some cells, kynurenine serves as an intermediate in the synthesis of NAD+, a coenzyme involved in numerous cellular reactions. A decrease in the level of methylnicotinamide, a degradation product of NAD+, was noted in the infected decidua and placenta extracts (Table 3). No change in methylnicotinamide was observed in the infected foetus.

The neurotransmitter serotonin, a derivative of tryptophan, was detected in the decidua using the C18-PFP column for the chromatography step; however, it was not found in the placental and foetal extracts under the same conditions. Significantly lower levels of serotonin were observed in the decidua from infected mice compared to the uninfected controls (Table 3).

### Metabolomic Analysis of Maternal Serum and Developing Foetus in Mice Infected With *T. gondii*

**FIGURE 5:**
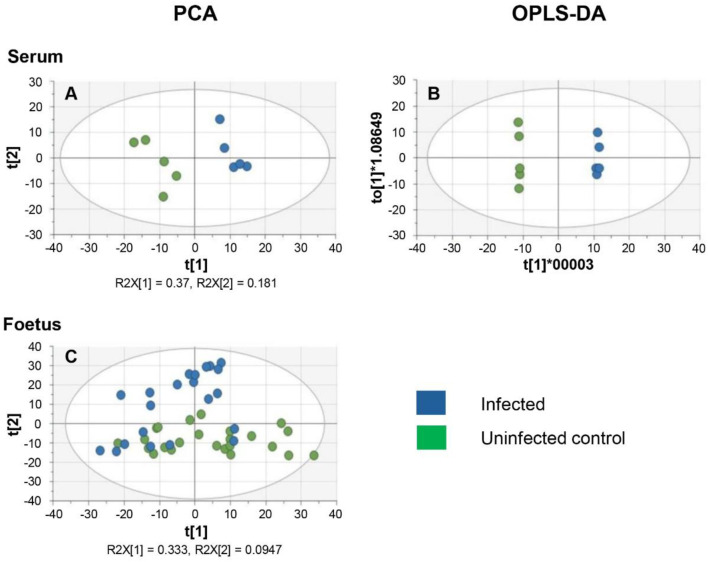
Multivariate analysis of the metabolism of serum and developing foetal extracts from *T. gondii*-infected day-13 pregnant mice (Infected; 6 days post-infection) or uninfected day-13 pregnant mice (Control) **(A)** PCA score plot of maternal serum extracts (n = 5 for each group). R2X[1] and R2X[2] indicate the proportion of the variance explained by the first and second components. **(B)** OPLS-DA plot of data from the maternal serum extracts. Each data point in **A** & **B** represents one mouse serum sample (n = 5, for each group of animals). **(C)** PCA score plot of the developing foetus shows a homogenous distribution of the data from the infected group (n = 22) and the control group (n = 24). It is worth noting that some samples derived from infected mothers cluster with samples from control mothers. Each data point represents a foetal extract.

In this study, the results previously obtained show that *T. gondii* infection induces similar changes in the decidua and placenta. However, the foetal extracts exhibited different results for the amino acids and some of their derivatives, including kynurenine and citrulline. To verify and extend these observations, a second experiment was carried out using a larger cohort of foetuses. Serum samples were analysed to determine changes in systemic maternal metabolism that could affect the foetus and the maternal-foetal interface. LCMS was carried out using the pHILIC column using experimental conditions that previously allowed the detection of the largest number of metabolites. Data from the PCA and OPLS-DA plots indicated a clear separation of the control and infected serum extracts (Figures 5A,B).

The PCA score plots for the foetal samples show a more diffuse distribution with the two groups overlapping (Figure 5C). OPLS-DA was therefore not a suitable model for the analysis of the foetal data. The inability of PCA to differentiate between foetuses from infected and uninfected mothers may be due to the fact that some foetuses from infected mothers might be infected with *T. gondii*, while others remain uninfected as previously reported [13].

**FIGURE 6:**
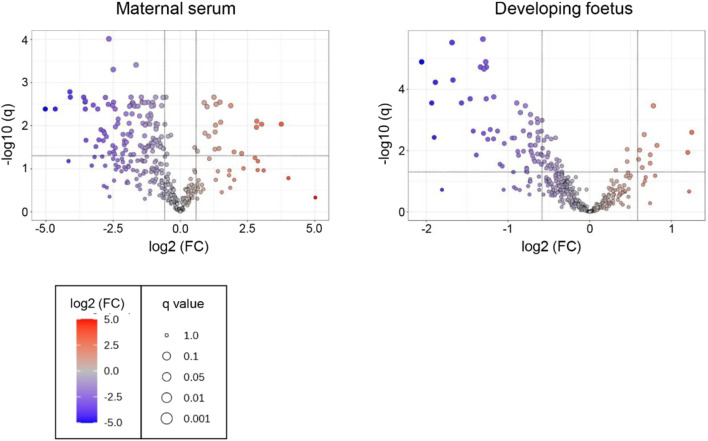
Volcano plots for the analysis of significant metabolites in the maternal serum and developing foetus from *T. gondii*-infected day-13 pregnant mice using the pHILLIC column. The X-axis shows log2 fold change with thresholds of −0.6 for a decreased level in infected extracts and of 0.6 for an increased level. The Y-axis is indicative of significance with the threshold set at −log10 q of 1.3. Significantly decreased or increased metabolites are represented by dots located in the upper left and upper right rectangles respectively. All other metabolites are below the threshold for significance.

Volcano plots were produced for the metabolites detected in both the serum and the foetal extracts (Figure 6). Table 4 shows the number of significantly altered metabolites identified in the maternal serum and developing foetus. The complete lists of metabolites identified with high confidence along with their formula, mass, retention time, log2 fold change and −log10 p and −log10 q values are included in the Supplementary Tables S8, S9. Significantly altered metabolites (log 2 FC ≤ −0.6 or ≥0.6, q ≥ 1.3). are indicated in these tables by shading.

**TABLE 4:** Numbers of metabolites identified in extracts from serum and foetus samples (second experiment).

Metabolite statistics	Serum	Foetus
All metabolites identified	368	421
Up	21	10
Down	110	67
Total dysregulated metabolites	131	77

All, total number of metabolites. Up, metabolites that showed significantly increased levels in extracts from Toxoplasma infected mice, compared with uninfected controls (Log2 FC, ≥0.6, −log10 q, ≥1.3). Down, metabolites that show decreased levels (log2 FC, ≤ −0.6, −log10 q, ≥1.3). Values shown for metabolites detected with the pHILIC column only, the C18-PFP column only or with both columns.

These volcano plots were used to generate a list of significantly altered (increased or decreased) metabolites (Supplementary Tables S5, S6).

### Significant Metabolic Changes Between the Developing Foetus and Maternal Serum

**FIGURE 7:**
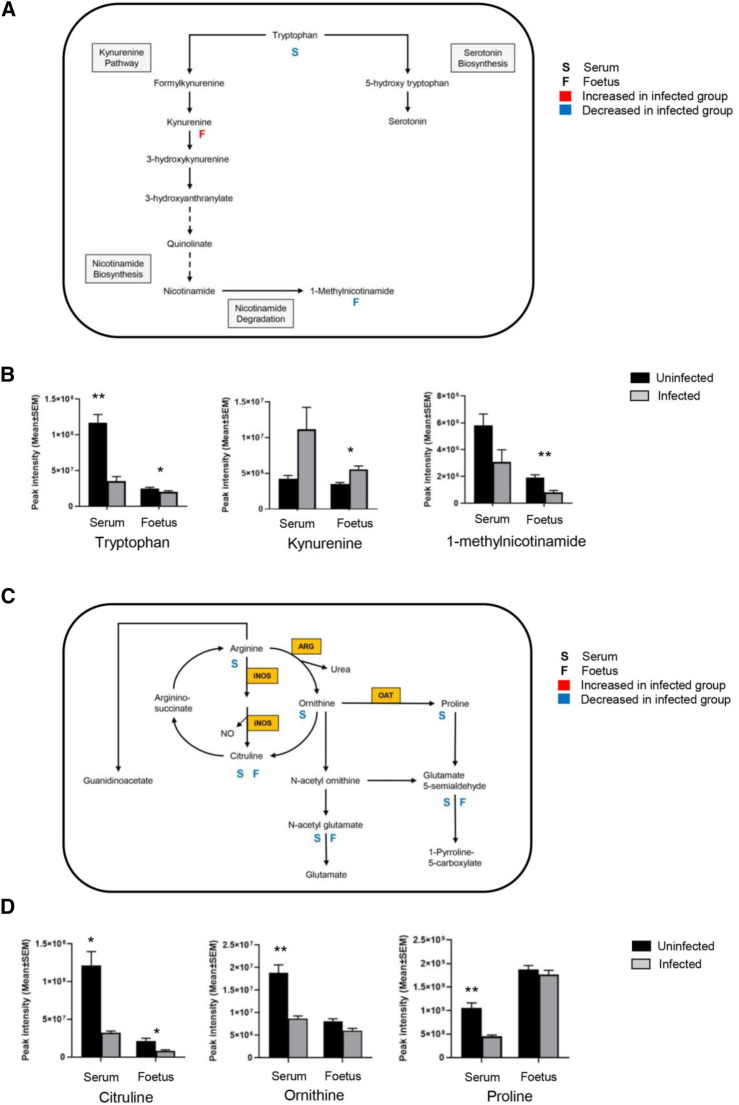
*T. gondii* infection results in altered tryptophan and arginine metabolism in serum and foetal extracts. **(A)** The tryptophan degradation pathway shows interactions between different metabolites in the tryptophan metabolic cycle, along with interactions with other pathways. Letter S below the rectangular box represents maternal serum; letter F is representative of the foetus. A significant log2 fold increase with a threshold of 0.6 is shown by red, and blue indicates a significant decrease of log2 fold change with a threshold of −0.6. **(B)** Levels of metabolites detected in the tryptophan degradation pathway in the serum from *T. gondii*-infected day-13 pregnant mice (infected, grey, n = 5) and uninfected day-13 pregnant animals (control, black, n = 5); and developing foetus from infected (n = 21) and control (n = 24) mice. Student’s t-test was performed to determine significance (−log10 q ≥ 1.3) where * -log10 q ≥ 1.3, ** −log10 q ≥ 2.3, *** −log10 q ≥ 3.3 are significant compared to the control. **(C)** The arginine metabolic pathway shows interactions between different metabolites along with interactions with other pathways. Enzymes catalysing key reactions: NOS, inducible nitric oxide synthase; ARG, arginase; OAT, ornithine amino transferase. **(D)** Levels of metabolites detected in the arginine metabolic pathway in the serum from infected (grey, n = 5) and control (black, n = 5); and developing foetus from infected (n = 21) and control (n = 24) mice. Student’s t-test was performed to determine significance (−log10 q ≥ 1.3) where * −log10 q ≥ 1.3, ** −log10 q ≥ 2.3, *** −log10 q ≥ 3.3 are significant compared to the control.

Most amino acids detected in the serum extracts were significantly downregulated in the infected group compared to the controls, as observed previously for the decidua (Table 5). With the exception of methionine, there was no change in the amino acid levels in the foetal extracts, confirming the results previously obtained. Kynurenine was significantly increased in the foetal extracts from *T. gondii*-infected day-13 pregnant mice (Table 6; Figures 7A,B), consistent with the results obtained with the previous set of foetal samples (Table 3). The foetal samples from *T. gondii*-infected day-13 pregnant mice had a decreased level of methylnicotinamide (Table 6), as also described for the decidua and placenta from *T. gondii*-infected day-14 pregnant mice. Serum samples from *T. gondii*-infected day-13 pregnant mice also showed increased kynurenine levels and decreased levels of 1-methylnicotinamide, but -log10 q values were just below the threshold for significance. Other intermediates in nicotinamide metabolism, such as nicotinate D-ribonucleoside (-log10 q 2.27) and N-ribosylnicotinamide (-log10 q 2.29), were decreased in the serum from *T. gondii*-infected day-13 pregnant mice.

**TABLE 5:** Dysregulation of protein amino acid levels in serum and foetus.

Metabolite	Serum		Foetus 2	
	log2 FC	−log10 q	log2 FC	−log10 q
**Arginine**	−0.81	**1.68**	−0.28	0.78
**Glutamate**	−1.37	0.89	−0.33	0.79
**Proline**	−1.21	**2.22**	−0.09	0.27
**Threonine**	−1.20	**1.51**	​	​
**Lysine**	−1.13	**2.14**	−0.06	0.08
**Methionine**	−1.31	**2.02**	−0.73	**3.19**
Tryptophan	−1.73	**2.48**	−0.30	0.92
**Tyrosine**	−0.89	0.88	−0.12	0.27
**Leucine**	−0.53	**2.64**	−0.30	0.81
**Valine**	−0.64	**2.61**	−0.13	0.74
**Alanine**	−0.84	**1.54**	−0.24	0.92
**Glutamine**	0.29	0.50	−0.02	0.02
**Phenylalanine**	0.10	0.28	0.28	0.68

Metabolite identities confirmed by matching retention time with an authentic standard are in bold type. FC, fold change (infected/control). Blue shading, more than 1.5 fold decrease in infected samples compared to controls (log2 FC ≤ −0.6). Statistically significant −log10 q values (−log10 q ≥ 1.3) are in bold type. Blank cells, metabolite not found in metabolomic profile. Data from LCMS using a pHILIC column for the chromatography step.

**TABLE 6:** Dysregulation of metabolic pathways in serum and foetus from toxoplasma infected mice.

Metabolite	Pathway	Serum		Foetus 2	
		log2 FC	−log10 q	log2 FC	−log10 q
**Lactate**	1	−1.02	**2.58**	−0.48	**1.50**
**Pyruvate**	1	−1.14	**2.03**	−0.59	**1.55**
**Citrate**	2	−1.59	1.19	−0.75	0.84
**Malate**	2	−2.41	**1.09**	−0.40	0.84
**Succinate**	2	−2.63	**1.36**	−0.13	0.27
**Hypoxanthine**	3	−2.41	**1,48**	−0.40	1.56
Xanthosine	3	−2.79	**2.64**	​	​
**Xanthine**	3	−1.31	**1.40**	​	​
**Urate**	3	1.08	1.23	​	​
**Kynurenine**	4	1.40	0.94	0.67	**3.52**
**Ornithine**	5, 6, 7, 8	−1.12	**2.26**	−0.42	1.18
**Citrulline**	5, 9	−1.91	**2.05**	−1.39	**1.86**
**Choline**	10	−1.10	**2.22**	−0.14	0.27
Homoarginine	11	−2.14	1.15	−0.71	**1.94**
**Methylnicotinamide**	12	−0.91	0.91	−1.22	**2.68**
Pipecolate^a^	13	−2.07	**1.61**	−0.78	**1.33**
Hippurate^a^	14	−4.09	**2.64**	−1.92	**3.54**
2-oxoarginine^a^	15	−1.49	**1.54**	−0.74	**2.53**
**Guanidinobutanoate^a^**	15	−2.17	1.24	−0.87	**1.85**
**Indoxylsulphate^a^**	16	−2.08	**1.65**	−1.90	**2.44**
TMAO^a^	17	​	​	−1.67	**4.30**

^a^ Production of metabolite is dependent on the gut microbiome.

Metabolite identities confirmed by matching retention time with an authentic standard are in bold type. FC, fold change (infected/control). Blue shading, more than 1.5 fold decrease in infected samples compared to controls (log2 FC ≤ −0.6); red shading, 1.5 fold increase in infected samples (log2 FC ≤ 0.6). Statistically significant −log10 q values (–log10 p ≥ 1.3) are in bold type. Values for significant differences are in bold. Blank cells, metabolite not found in metabolomic profile. Data from LCMS using a pHILIC column for the chromatography step. Pathways perturbed by infection: 1, glycolysis; 2, TCA cycle; 3, purine degradation degradation; 4, tryptophan degradation (kynurenine pathway); 5, arginine and proline metabolism; 6, urea cycle; 7, polyamine biosynthesis; 8, macrophage activation by T helper type 2 cytokines; 9, macrophage activation by T helper type 1 cytokines; 10, glycerophospholipid metabolism; 11, non-protein amino acid produced from arginine and lysine; 12, nicotimamide metabolism; 13, lysine degradation–gut microbiome dependent; 14, metabolism of aromatic compounds–gut microbiome dependent; 15, arginine metabolism–gut microbiome dependent; 16, tryptophan metabolism–gut microbiome dependent; 17, phosphatidylcholine, choline or carnitine metabolism–gut microbiome dependent.

The arginine metabolites detected in the serum showed similar changes to those already observed for the foetal tissues and at the maternal-foetal interface, with a significant decrease in the ornithine and citrulline levels (Table 6; Figures 7C,D). No information was obtained for spermine and spermidine because they were not detected with the pHILIC column. Citrulline levels were significantly reduced in the infected foetal extracts (-log10 q = 1.86) (Table 6; Figures 7C,D). There were decreased levels of ornithine, although this was not statistically significant. This observation agrees with the previous data for foetal extracts, decidua and placenta.

Serum samples showed changes in purine metabolism (Table 6) with significant reductions in xanthine (−log10 q = 1.40), hypoxanthine (−log10 q = 1.40), and xanthosine (−log10 q = 2.64). There were also increased levels of urate, but this was not significant (−log10 q = 1.23). A small decrease in hypoxanthine levels was observed in the infected foetal samples (−log10 q = 1.56), but no other purine metabolites were detected.

**FIGURE 8:**
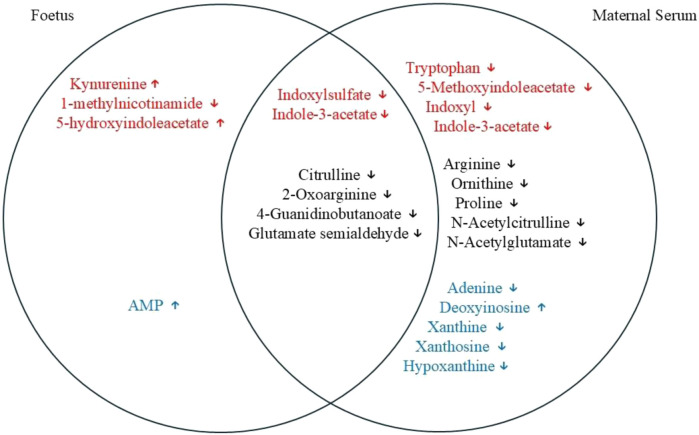
Venn diagram showing an overlap of different metabolites between maternal serum and the foetal extracts. Red font, tryptophan degradation pathway; black font, arginine and proline metabolism; and blue font, purine metabolism. Some of the metabolites can be seen as being altered significantly only in foetal extracts or in the maternal serum affected by *T. gondii*. Some of the metabolites that are changed significantly in both of the tissues examined are also shown in the diagram.

The results obtained here identified various metabolites that, through association, highlight tryptophan degradation, arginine metabolism and purine degradation as the metabolic pathways most affected by the infection. Notably, most metabolic changes were found in the maternal serum, but a subset of these were also found to be affected in the foetus. The Venn diagram shows an overlap of various metabolites, a few of which were only noted to change in the foetus (Figure 8).

### *T. gondii* Infection Modifies the Host’s Microbiome

**FIGURE 9:**
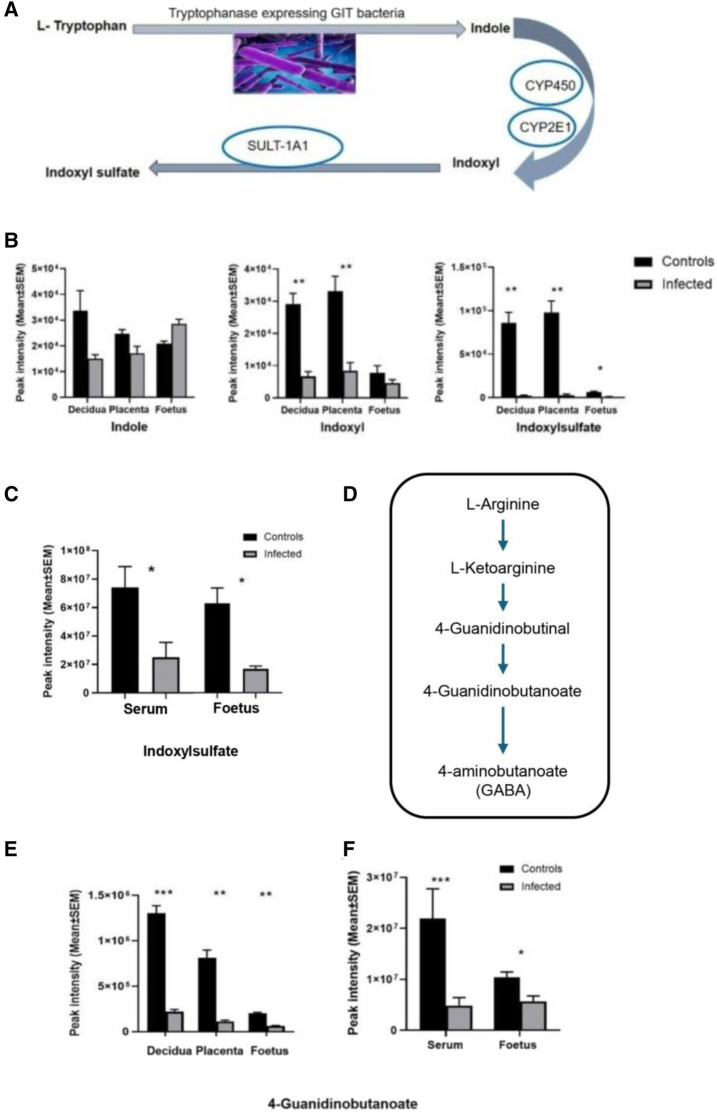
**(A)** The origin of indoxyl and indoxylsulfate (IS) is due to tryptophan utilisation by tryptophanase-expressing bacteria in the gut. Conversion of tryptophan to indole takes place in the gut by the microbiome activity, and indole is converted to indoxyl by the liver’s enzymatic reactions (mainly cytochromes, CYP450, CYP2E1). Then it is converted to indoxylsulfate by the sulfotransferase enzyme (SULT-1A1). **(B)** Changes in the levels of indole, indoxyl and indoxyl sulfate detected in this study. Decidual and placental extracts were obtained from uninfected day-14 pregnant mice (n = 10) and infected day-14 pregnant mice (n = 10). Foetal extracts were from uninfected day-14 pregnant mice (n = 14) and infected day-14 pregnant mice (n = 8). Graphs represent the mean peak intensity of the metabolite ±SEM. Student’s t-test was performed to determine significance (−log10 q ≥ 1.3) where * −log10 q ≥ 1.3, ** -log10 q ≥ 2.3, are significant compared to the control. **(C)** Represents metabolite indoxylsulfate detected in the maternal serum and the developing foetus. Maternal serum extracts (n = 5 for uninfected day-13 pregnant mice and infected day-13 pregnant mice). Foetal extracts (n = 24 and 21 for uninfected day-13 pregnant mice and infected day-13 pregnant mice, respectively). The graphs represent the mean peak intensity of indoxylsulfate in each of the tissues. **(D)** Arginine catabolic pathway in *Pseudomonas aeruginosa*. Origin of 4-guanidinobutanoate as a result of *P. aeruginosa* (PAO1) reaction in the L-arginine metabolic pathway. **(E)** Microbial metabolite 4-guanidinobutanoate detected in the decidual and placental extracts (n = 13 and n = 10 for uninfected day-14 pregnant mice and infected day-14 pregnant mice, respectively). Foetal extracts (n = 14 and n = 8 for uninfected day-14 pregnant mice and infected day-14 pregnant mice, respectively). **(F)** 4-guanidinobutanoate detected in the maternal serum and the developing foetus. Maternal serum extracts (n = 5 for uninfected day-13 pregnant mice and infected day-13 pregnant mice). Foetal extracts (n = 24 and 21 for uninfected day-13 pregnant mice and infected day-13 pregnant mice, respectively). For graphs in **B** and **E**, data was obtained from experiment 1 (decidua, placenta and foetus). Data for C and F was obtained from experiment 2 (foetus and maternal serum). For all graphs, black bars show metabolite mean peak intensity (±SEM) from unifected controls and grey bars show values from infected animals. Student’s t-test was performed to determine significance (-log10 q ≥ 1.3) where * -log10 q ≥ 1.3, ** -log10 q ≥ 2.3, *** -log10 q ≥ 3.3 are significant compared to the control uninfected.

The global metabolomic analysis identified some metabolites from microbiome origin. These were found to be altered in the serum, as well as in the decidua, placenta, and the developing foetuses of the mice infected with *T. gondii*. These microbial metabolites, including indoxylsulfate and 4-guanidinobutanoate, are reliant on the metabolism of the microbiome for their production. Indoxylsulfate is a microbiome-host co-metabolite (Figure 9A). First, indole is produced from tryptophan by tryptophanase-expressing bacteria in the gut. Then, indole is absorbed into the bloodstream and converted to indoxyl and indoxylsulfate in the liver. *T. gondii* infection resulted in significantly lower levels of indoxylsulfate in the decidua, placenta and foetus (Figure 9B; Table 3). Decreased levels of indoxylsulfate were also seen in the infected maternal serum compared with the control mice (Figure 9C; Table 6). The biosynthesis of guanidinobutanoate occurs in the presence of *Pseudomonas aeruginosa* in the arginine catabolic pathway (Figure 9D). There was a significant reduction of 4-guanidinobutanoate in the decidua, placenta and developing foetus Figure 9E; Table 3). The 4-guanidinobutanoate was significantly reduced in foetal extracts from infected mice, confirming previous results and was also reduced in infected maternal serum (Figure 9F; Table 6). Arginine levels were lower in the maternal serum and 2-oxoarginine, an intermediate in the production of 4-guanidinobutanoate from arginine, was also found to be lower in infected sera and foetal extracts (Table 6). TMAO, a microbiome-dependent derivative of choline, was present at reduced levels in the infected foetal extracts (Table 6). It was not detected in the serum samples, but choline was significantly decreased in the infected serum and foetus (Table 6). *T. gondii* infection resulted in reduced levels of other microbiome dependent metabolites in the maternal sera and devoloping foetus, these included hippurate and pipecolate (Table 6).

## Discussion

Studying the effect of *T. gondii* infection on the maternal-foetal interface is important as it could contribute to our understanding of maternal and foetal outcomes due to *T. gondii* infection and ultimately the development of treatments to minimise its impact. Furthermore, elucidating early events in the foetus could lead to treatments to reduce the effects of congenital infection. Although some recent studies have focused on studying the immune events at the maternal-foetal interface using the murine model [16], no studies to date have examined changes to metabolism in the maternal-foetal interface and developing foetus. Metabolism is now known to be bi-directionally linked to the immune response. However, the importance of this at the maternal-foetal interface, and how infectious diseases affect it, merits further study. Metabolism at the maternal-foetal interface is an even less studied issue. Logically, the interplay between the immune response and metabolism at this interface could be disrupted directly by the metabolic demands of microbes, damage to the decidua or placenta, or indirectly by the immune response they induce. The consequences could range from disrupted pregnancies to developmental disorders, due to the disruption of nutritional needs or the transplacental transfer of toxic metabolites. The studies described here demonstrate that *T. gondii* infection affects the metabolism at the maternal-foetal interface and the metabolites found in the developing foetus. The full consequences of these observations are not yet clear.

Initially, a global LC-MS based metabolomics approach was used to determine how metabolites are altered in the maternal-foetal interface and the developing foetus extracted from the mothers infected with *T. gondii* infection. Biological tissues exploited in pregnancy were placenta, decidua, developing foetus and maternal serum. The results indicate various changes in the metabolomic profile of these tissues taken from the mothers infected with *T. gondii.* Previous studies have characterised changes in sera from *T. gondii-*infected mice. Although different platforms were used for LCMS, Supplementary Table S10 shows that where the metabolomic profiles overlap, the results obtained for maternal sera in this study are in very good agreement [17, 18]. One notable feature was the dysregulation of serum amino acid homeostasis, with many of the amino acids showing significantly lower levels in the infected sera. In this study, the same effect was seen in the decidua of infected mice, whereas the foetal extracts showed no change.

Interestingly, the metabolites that showed a significant decrease were citrulline and arginine succinate, while spermine and spermidine were increased significantly. Proline was significantly reduced in the maternal sera, decidua and placenta. L-arginine was significantly reduced in the sera and decidual extracts, while no change was noted in the foetal extracts taken from the mothers infected with *T. gondii*. During pregnancy, dietary intake of arginine is essential as it is a semi-essential amino acid [19]. Other than being used as a building block for the synthesis of proteins, arginine is also used as a substrate for important metabolic pathways affecting macrophages, dendritic cells and T cells immune characters [20]. Arginine is a precursor for important biologically active compounds, including nitric oxide (NO), polyamines, creatine and phosphocreatine [21]. Lower levels of the arginine metabolites citrulline and argininosuccinate were seen in decidua, placenta and foetal tissue from infected mice while spermidine and spermidine increased. Our results are suggestive of maternal arginine degradation being utilised for the synthesis of polyamines as increased levels of polyamines were noted following infection. Another possibility to explain the decreased levels of maternal arginine and citrulline during infection might be a change to the intestinal-renal axis, where *de novo* synthesis of citrulline takes place from glutamate [22]. In support of this, decreased levels of L-glutamate and N-acetyl-glutamate were also recorded. Reduced intake of arginine/citrulline due to protein malnutrition as well as depletion of endogenous L-arginine due to the maternal infection, e.g., malaria, have been associated with pre-eclampsia and poor pregnancy outcomes [23]. Conversely, arginine and citrulline supplementation have been shown to be beneficial for pregnancy outcome [24]. Thus, the observation that *T. gondii* infection also reduces arginine levels raises the possibility that a similar treatment during pregnancy might alleviate some of the detrimental effects of infection.

Tryptophan metabolism can be categorised into endogenous and bacterial. The data presented here demonstrate that *T. gondii* induces changes to both of these metabolic processes. Kynurenine is one of the endogenous metabolites of kynurenine pathway (KP) of tryptophan degradation [25, 26]. Kynurenine pathway is initiated by the IDO and TDO enzymes that are stimulated by inflammatory cytokines and IFN-γ [27]. The evaluation of the tryptophan degradation pathway indicated that some metabolites, including kynurenine and tryptophan, had changed. While kynurenine was downregulated in the decidua and placenta of infected mice relative to control mice, it was upregulated in the foetal extracts. L-tryptophan was decreased only in the decidual extracts of infected mice relative to control mice. Interaction of the tryptophan degradation pathway with the nicotinate pathway resulted in a significant decrease of 1-methylnicotinamide in all of the tissues examined from infected mice relative to control mice. Notably, levels of tryptophan were decreased in the maternal serum of *T. gondii* infected mice. *T. gondii* infection was previously shown to increase kynurenine levels in female mouse sera. The same trend was observed in this study, although the difference was not statistically significant. These results demonstrate that *T. gondii* infection increases the tryptophan degradation pathway towards the kynurenine pathway rather than the serotonin pathway. This results in increased levels of kynurenine in developing foetuses. Notably, kynurenine has been found to cross the placenta [28]. Therefore, the increased levels detected in the foetuses could be derived from the maternal immune response to *T. gondii,* which is known to induce indolamine deoxygenase expression and degradation of tryptophan [29]. This could have implications for the health of the offspring as foetal exposure to kynurenine has been linked with increased risk of psychoneurological disease later in life [28, 30, 31].

Overall, all these changes observed allowed us to conclude that the maternal infection with *T. gondii* has the ability to affect the foetus at a very early stage.

Our studies also described that *T. gondii* infection alters bacterial tryptophan metabolism. Thus, the level of indoxylsulfate, a metabolite of bacterial origin from tryptophan degradation**,** was changed in infected mice. Lactobacilli/tryptophanase producing bacterial species can metabolize tryptophan to produce indole [32]. Levels of indoxylsulfate were noted as decreased following maternal *T. gondii* infection, with a statistical significance in all of the tissues examined. Cytochrome P450 enzymes are responsible for the oxidation of indole to indoxyl, which acts as an intermediate for the synthesis of indoxylsulfate [33]. This is a protein bound uremic toxin that is a metabolite of the microbiome produced from protein fermentation of the microbial population residing in the gut microbiota. A considerable amount of literature associates high concentrations of indoxylsulfate with renal insufficiency, and it is considered an important biomarker for chronic kidney disease [34–36]. Some toxic effects of indoxylsulfate include stimulation of inflammation and immune responses by upregulation of the MCP-1 and leukocyte interactions.

The study also covered additional metabolite changes associated with the microbiome, mainly 4-guanidinobutanoate (4-GB) and hippurate. In this study 4-Guanidinobutanoate (4-GB) levels were found to be decreased in all of the examined tissues of infected animals relative to control animals (developing foetus, decidua, placenta and maternal serum). It is produced by two related pathways in *P. aeruginosa*, the D-arginine dehydrogenase pathway [37, 38] and the L-arginine transaminase pathway [39]*.* 2-oxoarginine, a common intermediate in both pathways, was present at significantly lower levels in maternal serum and foetal extracts, providing further evidence that these pathways are deregulated. 4-GB is also a metabolite of GABA formed by the transfer of the amidino group from arginine to the amino group of GABA, in a reaction catalysed by the multifunctional glycine amidinotransferase in mice and humans [40]. In other studies, some indications of GABAergic effects of this metabolite have been reported, which is presumably considered an important metabolite for neural functions [41, 42]. Hippurate is an abundant host-microbiome co-metabolite produced in the liver through the conjugation of glycine and benzoate, which is derived from the bacterial catabolism of aromatic compounds. Low hippurate levels have been associated with reduced microbiome gene richness and poorer metabolic health [43]. The result in the current study suggests that *T. gondii* induces changes in the microbiome. However, the implications of this change for the outcome of pregnancy and foetal development remain to be determined.

Overall, the results described here demonstrate, for the first time, that *T. gondii* induces changes in metabolites at the maternal-foetal interface and in the developing foetus. More widely, the model used and these initial findings lay the groundwork for further studies that could focus on the implications of these findings for pregnancy and life-long health of foetus, and ultimately adult offspring. The most striking finding is that kynurenine, a metabolite that has been associated with the development of psychoneurological disease, is raised in the foetus at early/mid stages of gestation. This finding could have practical use, as it suggests that developing treatments to reduce kynurenine production and their application early in life could have an impact on the prevention of psychoneurological diseases later in life.

## Summary Table

### What Is Known About This Subject

- Infection with *Toxoplasma gondii* during pregnancy can result in abortion or congenital infection
- The maternal-foetal interface forms a selective barrier between the maternal and foetal circulations and immunological events occurring are essential for successful pregnancy and *T. gondii*
- *T. gondii* infection is known to influence immunological events and alter host metabolism in other tissues of adult mice

### What This Paper Adds

- Through use of liquid chromatography mass spectrometry (LCMS) in the BALB/c murine model of congenital *T. gondii* we demonstrate *T. gondii* induced changes to metabolism, including amino acids, urea cycle in maternal serum, the decidua, placenta and foetus
- Indoxylsulfate and 4-guanidinobutanoate, metabolites from microbiome origin were altered in maternal and foetal tissues of *T. gondii-infected mice*
- A significant increase in kynurenine levels was evident in foetal extracts from *T. gondii*-infected mice.

### Concluding Statement

This work represents an advance in biomedical science because it demonstrates *T. gondii*-induced metabolite changes in, maternal serum, the foetal/maternal interface and an increase in kynurenine in the developing foetus.

## Data Availability

Data generated are available at https://doi.org/10.15129/b313b637-48e3-46bb-bd37-3a25f396274d.

## References

[1] Hampton MM . Congenital Toxoplasmosis: A Review. Neonatal Netw (2015) 34(5):274–8. 10.1891/0730-0832.34.5.274 26802827

[2] Abbasi M Kowalewska-Grochowska K Bahar MA Kilani RT Winkler-Dunn D Wallon M Mother-to- Child Transmission of Toxoplasmosis: Risk Estimates for Clinical Counselling. Lancet (1999) 353(9167):1829–33. 10359407 10.1016/S0140-6736(98)08220-8

[3] Dunn D Wallon M Peyron F Petersen E Peckham C Gilbert R . Mother-To-Child Transmission of Toxoplasmosis: Risk Estimates for Clinical Counselling. Lancet (1999) 353(9167):1829–33. 10.1016/S0140-6736(98)08220-8 10359407

[4] Robert-Gangneux F Dardé ML . Epidemiology of and Diagnostic Strategies for Toxoplasmosis. Clin Microbiol Rev (2012) 25(2):264–96. 10.1128/CMR.05013-11 22491772 PMC3346298

[5] Roberts C Alexander J . Studies on a Murine Model of Congenital Toxoplasmosis: Vertical Disease Transmission Only Occurs in BALB/c Mice Infected for the First Time During Pregnancy. Parasitology (1992) 104(1):19–23. 10.1017/s0031182000060753 1614736

[6] Bollani L Strocchio L Stronati M . Congenital Toxoplasmosis. Early Hum Dev (2013) 89(Suppl. 1):S70–2. 10.1016/s0378-3782(13)70107-5 23809350

[7] Dubey JP . Toxoplasmosis - a Waterborne Zoonosis. Vet Parasitol (2004) 126(1-2):57–72. 10.1016/j.vetpar.2004.09.005 15567579

[8] Kafsack BF Llinás M . Eating at the Table of Another: Metabolomics of Host-Parasite Interactions. Cell Host Microbe (2010) 7(2):90–9. 10.1016/j.chom.2010.01.008 20159614 PMC2825149

[9] Hargrave KE Woods S Millington O Chalmers S Westrop GD Roberts CW . Multi-Omics Studies Demonstrate Toxoplasma gondii-Induced Metabolic Reprogramming of Murine Dendritic Cells. Front Cell Infect Microbiol (2019) 9:309. 10.3389/fcimb.2019.00309 31572687 PMC6749083

[10] Blume M Seeber F . Metabolic Interactions Between Toxoplasma gondii and Its Host. F1000Res (2018) 7:F1000 Faculty Rev-1719. 10.12688/f1000research.16021 30467519 PMC6208699

[11] Coppens I . Exploitation of Auxotrophies and Metabolic Defects in Toxoplasma as Therapeutic Approaches. Int J Parasitol (2014) 44(2):109–20. 10.1016/j.ijpara.2013.09.003 24184910

[12] Kramer AC Jansson T Bale TL Powell TL . Maternal-Fetal Cross-Talk via the Placenta: Influence on Offspring Development and Metabolism. Development (2023) 150(20):dev202088. 10.1242/dev.202088 37831056 PMC10617615

[13] Brito C Silva TM Castro MA Wyrwas W Oliveira BM Fonseca BM Toxoplasma gondii Infection Reduces Serum Progesterone Levels and Adverse Effects at the Maternal-Foetal Interface. Parasite Immunol (2020) 42(2):e12690. 10.1111/pim.12690 31802508

[14] Fonseca BM Correia-da-Silva G Taylor AH Konje JC Bell SC Teixeira NA . Spatio-Temporal Expression Patterns of Anandamide-Binding Receptors in Rat Implantation Sites: Evidence for a Role of the Endocannabinoid System During the Period of Placental Development. Reprod Biol Endocrinol (2009) 7:121. 10.1186/1477-7827-7-121 19860893 PMC2775033

[15] Creek DJ Jankevics A Burgess KE Breitling R Barrett MP . IDEOM: An Excel Interface for Analysis of LC-MS-Based Metabolomics Data. Bioinformatics (2012) 28(7):1048–9. 10.1093/bioinformatics/bts069 22308147

[16] Prabhudas M Bonney E Caron K Dey S Erlebacher A Fazleabas A Immune Mechanisms at the Maternal-Fetal Interface: Perspectives and Challenges. Nat Immunol (2015) 16(4):328–34. 10.1038/ni.3131 25789673 PMC5070970

[17] Zhou C Zhou D Elsheikha H Zhao Y Suo X Zhu X . Metabolic Profiling of Mice Serum During Toxoplasmosis Progression Using Liquid Chromatography-Mass Spectrometry. Sci Rep (2016) 6:19557. 10.1038/srep19557 26785939 PMC4726199

[18] Zhou C Cong W Chen X He S Elsheikha H Zhu X . Serum Metabolic Profiling of Oocyst-Induced Toxoplasma gondii Acute and Chronic Infections in Mice Using Mass Spectrometry. Front Microbiol (2017) 8:2512. 10.3389/fmicb.2017.02612 29354104 PMC5761440

[19] Hsu CN Tain YL . Impact of Arginine Nutrition and Metabolism During Pregnancy on Offspring Outcomes. Nutrients (2019) 11(6):1452. 10.3390/nu11071452 31252534 PMC6682918

[20] Kim SH Roszik J Grimm EA Ekmekcioglu S . Impact of L-Arginine Metabolism on Immune Response and Anticancer Immunotherapy. Front Oncol (2018) 8:67. 10.3389/fonc.2018.00067 29616189 PMC5864849

[21] Martí ILA Reith W . Arginine-Dependent Immune Responses. Cell Mol Life Sci (2021) 78(15):5303–24. 10.1007/s00018-021-03828-4 34037806 PMC8257534

[22] Dhanakoti SN Brosnan JT Herzberg GR Brosnan ME . Renal Arginine Synthesis: Studies *In Vitro* and *In Vivo* . Am J Physiol (1990) 259(3 Pt 1):E437–42. 10.1152/ajpendo.1990.259.3.E437 1975989

[23] McDonald CR Cahill LS Gamble JL Elphinstone R Gazdzinski LM Zhong KJY Malaria in Pregnancy Alters L-Arginine Bioavailability and Placental Vascular Development. Sci Transl Med (2018) 10(431):eaao9375. 10.1126/scitranslmed.aan6007 29514999 PMC6510298

[24] Weckman AM McDonald CR Baxter JB Fawzi WW Conroy AL Kain KC . Perspective: L-Arginine and L-Citrulline Supplementation in Pregnancy: A Potential Strategy to Improve Birth Outcomes in Low-Resource Settings. Adv Nutr (2019) 10(5):765–77. 10.1093/advances/nmz015 31075164 PMC6743852

[25] Chen Y Guillemin GJ . Kynurenine Pathway Metabolites in Humans: Disease and Healthy States. Int J Tryptophan Res (2009) 2:1–19. 10.4137/ijtr.s2097 22084578 PMC3195227

[26] Savitz J . The Kynurenine Pathway: A Finger in Every Pie. Mol Psychiatry (2020) 25(1):131–47. 10.1038/s41380-019-0414-4 30980044 PMC6790159

[27] Sordillo PP Sordillo LA Helson L . The Kynurenine Pathway: A Primary Resistance Mechanism in Patients with Glioblastoma. Anticancer Res (2017) 37(5):2159–71. 10.21873/anticanres.11551 28476779

[28] Goeden N Notarangelo FM Pocivavsek A Beggiato S Bonnin A Schwarcz R . Prenatal Dynamics of Kynurenine Pathway Metabolism in Mice: Focus on Kynurenic Acid. Dev Neurosci (2017) 39(6):519–28. 10.1159/000481168 29080891 PMC6338215

[29] Gómez-Chávez F Cañedo-Solares I Ortiz-Alegría LB Flores-García Y Luna-Pastén H Figueroa-Damián R Maternal Immune Response During Pregnancy and Vertical Transmission in Human Toxoplasmosis. Front Immunol (2019) 10:285. 10.3389/fimmu.2019.00285 30846989 PMC6393384

[30] Dantzer R . Role of the Kynurenine Metabolism Pathway in Inflammation-Induced Depression: Preclinical Approaches. Curr Top Behav Neurosci (2017) 31:117–38. 10.1007/7854_2016_6 27225497 PMC6585430

[31] Li H Liu T Heinsberg LW Lockwood MB Wainwright DA Jang MK Systematic Review of the Kynurenine Pathway and Psychoneurological Symptoms Among Adult Cancer Survivors. Biol Res Nurs (2020) 22(4):472–84. 10.1177/1099800420938141 32602357 PMC7708728

[32] Roager HM Licht TR . Microbial Tryptophan Catabolites in Health and Disease. Nat Commun (2018) 9:3294. 10.1038/s41467-018-05470-4 30120222 PMC6098093

[33] Banoglu E Jha GG King RS . Hepatic Microsomal Metabolism of Indole to Indoxyl, a Precursor of Indoxyl Sulfate. Eur J Drug Metab Pharmacokinet (2001) 26(3):235–40. 10.1007/BF03226377 11808865 PMC2254176

[34] Sun CY Hsu HH Wu MS . p-Cresol Sulfate and Indoxyl Sulfate Induce Similar Cellular Inflammatory Gene Expressions in Cultured Proximal Renal Tubular Cells. Nephrol Dial Transpl (2013) 28(1):70–8. 10.1093/ndt/gfs133 22610984

[35] Tan X Cao X Zou J Shen B Zhang X Liu Z Indoxyl Sulfate, a Valuable Biomarker in Chronic Kidney Disease and Dialysis. Hemodial Int (2017) 21(2):161–7. 10.1111/hdi.12483 27616754

[36] Gryp T De Paepe K Vanholder R Kerckhof FM Van Biesen W Van de Wiele T Gut Microbiota Generation of Protein-Bound Uremic Toxins and Related Metabolites Is Not Altered at Different Stages of Chronic Kidney Disease. Kidney Int (2020) 97(6):1230–42. 10.1016/j.kint.2020.01.028 32317112

[37] Jann A Matsumoto H Haas D . The Fourth Arginine Catabolic Pathway of *Pseudomonas aeruginosa* . J Gen Microbiol (1988) 134(5):1043–53. 10.1099/00221287-134-4-1043 3141581

[38] Nakada Y Itoh Y . *Pseudomonas aeruginosa* PAO1 Genes for 3-guanidinopropionate and 4-guanidinobutyrate Utilization May Be Derived from a Common Ancestor. Microbiology (Reading) (2005) 151(12):4055–62. 10.1099/mic.0.28258-0 16339950

[39] Wei Y Ding J Li J Cai S Liu S Hong L Metabolic Reprogramming of Immune Cells at the Maternal-Fetal Interface and the Development of Techniques for Immunometabolism. Front Immunol (2021) 12:717014. 10.3389/fimmu.2021.717014 34566973 PMC8458575

[40] Meera P Uusi-Oukari M Wallner M Libschutz G . Guanidinoacetate (GAA) Is a Potent GABAA Receptor GABA Mimetic: Implications for Neurological Disease Pathology. J Neurochem (2023) 165(4):445–54. 10.1111/jnc.15774 36726215

[41] Adkins DE McClay JL Vunck SA Batman AM Vann RE Clark SL Behavioral Metabolomics Analysis Identifies Novel Neurochemical Signatures in Methamphetamine Sensitization. Genes Brain Behav (2013) 12(7):780–91. 10.1111/gbb.12081 24034544 PMC3922980

[42] Watson DG Pomeroy PP Al-Tannak NF Kennedy MW . Stockpiling by Pups and Self-Sacrifice by Their Fasting Mothers Observed in Birth to Weaning Serum Metabolomes of Atlantic Grey Seals. Sci Rep (2020) 10:7465. 10.1038/s41598-020-64488-1 32366923 PMC7198541

[43] Brial F Chilloux J Nielsen T Vieira-Silva S Falony G Andrikopoulos P Human and Preclinical Studies of the Host–Gut Microbiome Co-Metabolite Hippurate as a Marker and Mediator of Metabolic Health. Gut (2021) 70(11):2105–14. 10.1136/gutjnl-2020-323314 33975870 PMC8515120

[44] Arshad H . Metabolomic Profiling of Maternal–Fetal Interface During Toxoplasma Gondii Infection [PhD thesis]. Glasgow: University of Strathclyde (2022).

