# Supplementary material for: Maternal Toxoplasma gondii Infection Perturbs Foetal and Maternal Foetal Interface Metabolism, Exposing the Foetus to Kynurenine
Source: Br J Biomed Sci. 2026 Feb 4;82:14989. doi: 10.3389/bjbs.2025.14989 (PMC12913195; doi:10.3389/bjbs.2025.14989)
Supplement: Supplementary file 3 [file Table1.docx]

| Exp. | Group | Day of Pregnancy | Days Post Infection | Decidua  Samples | Placenta  Samples | Foetus  Samples |
| --- | --- | --- | --- | --- | --- | --- |
| #1 | Control (n = 2) | 14 | - | 13 | 13 | 14 |
|  | Infected (n = 2) | 14 | 7 | 10 | 10 | 8 |
| #2 | Control (n = 6) | 13 | - | - | - | 24 |
|  | Infected (n = 7) | 13 | 6 | - | - | 21 |

**Table S1. Animal groups used in this study and tissue samples collected for LCMS.** In this study, four groups of mice were used as follows: mice at day 13 of pregnancy uninfected (control); mice at day 13 of pregnancy and 6 days of infection; mice at day 14 of pregnancy uninfected (control); and mice at day 14 of pregnancy and 7 days of infection. Sera was collected from animals of Experiment #2.
