# Supplementary material for: Maternal Toxoplasma gondii Infection Perturbs Foetal and Maternal Foetal Interface Metabolism, Exposing the Foetus to Kynurenine
Source: Br J Biomed Sci. 2026 Feb 4;82:14989. doi: 10.3389/bjbs.2025.14989 (PMC12913195; doi:10.3389/bjbs.2025.14989)
Supplement: Supplementary file 7 [file Table11.docx]

| Exp. | Group | Day of Pregnancy | Days Post Infection | Decidua  Weight (mg) | Placenta  Weight (mg) | Foetus  Weight (mg) |
| --- | --- | --- | --- | --- | --- | --- |
| #1 | Control (n = 2) | 14 | - | 43±17 | 66±30 | 97±28 |
|  | Infected (n = 2) | 14 | 7 | 39±8 | 53±7 | 117±20 |
| #2 | Control (n = 6) | 13 | - | - | - | 68±36 |
|  | Infected (n = 7) | 13 | 6 | - | - | 56±16 |

**Table S11. Tissue weight of the tissues collected from mice for LC-MS analysis.** In this study, four groups of mice were used as follows: mice at day 13 of pregnancy uninfected (control); mice at day 13 of pregnancy and 6 days of infection; mice at day 14 of pregnancy uninfected (control); and mice at day 14 of pregnancy and 7 days of infection. Sera was collected from animals of Experiment #2.
