# Supplementary material for: Maternal Toxoplasma gondii Infection Perturbs Foetal and Maternal Foetal Interface Metabolism, Exposing the Foetus to Kynurenine
Source: Br J Biomed Sci. 2026 Feb 4;82:14989. doi: 10.3389/bjbs.2025.14989 (PMC12913195; doi:10.3389/bjbs.2025.14989)
Supplement: Supplementary file 11 [file Table10.docx]

| Metabolite | This study | | | Zhou *et al.* 2015 | | Zhou *et al.* 2018 | |
| --- | --- | --- | --- | --- | --- | --- | --- |
|  | log2 FC | -log10 p | -log10 q | log2 FC | -log10 p | log2 FC | -log10 q |
| Citrate | -1.59 | **1.60** | 1.19 | 1.21 | **4.30** |  |  |
| Malate | -2.41 | **1.46** | 1.09 | -0.69 | **1.96** |  |  |
| Fumarate | -1.92 | **1.40** | 1.04 | -0.77 | **2.95** |  |  |
| Succinate | -2.63 | **1.83** | **1.36** |  |  |  |  |
| Arginine | -0.81 | **1.68** | **2.30** | -1.63 | **> 1.3** | -1.63 | **1.87** |
| Tryptophan | -1.73 | **3.60** | **2.48** | -1.60 | **> 1.3** | -2.38 | **2.44** |
| Tyrosine | -0.89 | 0.88 | 1.17 | -1.23 | **> 1.3** | -3.11 | **2.87** |
| Phenylalanine | 0.10 | 0.37 | 0.28 |  |  | 0.39 | **2.35** |
| Glutamine | 0.29 | 0.67 | 0.50 |  |  | 0.74 | **1.07** |
| Methionine | -1.31 | **2.77** | **2.02** | -0.35 | **> 1.3** |  |  |
| Leucine | -0.53 | **4.34** | **2.64** | -0.51 | **> 1.3** |  |  |
| Valine | -0.64 | **4.06** | **2.61** | -0.65 | **> 1.3** |  |  |
| Threonine | -1.20 | **2.05** | **1.51** |  |  |  |  |
| Proline | -1.21 | **2.22** | **3.10** |  |  |  |  |
| Lysine | -1.13 | **2.99** | **2.14** |  |  |  |  |
| Alanine | -0.84 | **2.10** | **1.54** |  |  |  |  |
| Citrulline | -1.91 | **2.87** | **2.05** | -1.94 | **> 1.3** | -1.94 | **1.74** |
| Ornithine | -1.12 | **3.15** | **2.26** |  |  |  |  |
| Kynurenine | 1.40 | **1.25** | 0.94 | 1.77 | **3.81** |  |  |
| Choline | -1.10 | **3.09** | **2.22** | -0.93 | **6.22** |  |  |
| Orotate | -1.23 | **2.84** | **2.04** |  |  | Up * |  |
| Uric acid | 1.08 | **1.66** | 1.23 |  |  | Up * |  |
| Hypoxanthine | -2.41 | **1.99** | **1.48** |  |  | Down * |  |

**Table S10. Dysregulation of metabolic pathways in serum samples from toxoplasma infected mice.** Comparison of results from three recent studies. FC, fold change (infected/control). Blue shading, more than 1.5 fold decrease in infected samples compared to controls (log2 FC ≤ -0.6); red shading, 1.5 fold increase in infected samples (log2 FC ≤ 0.6). Statistically significant -log10 q values ( –log10 p ≥ 1.3) are in bold type. Values for significant differences are in bold. Blank cells, no data reported for the metabolite. Values for significant differences are in bold. *Values for log2 FC and –log10 q not given in the publication.
